# Reduced Expression of Oestrogen Receptors Alpha and Beta in the Prepuce and Urethral Plate of Hypospadias Patients: A Clinical Study

**DOI:** 10.7759/cureus.87567

**Published:** 2025-07-08

**Authors:** Vijayta Singh, Suresh Yadav, Vivek K Singh, Vishwajeet Singh, Ajay R Handa, Krishna Bhandari

**Affiliations:** 1 Pathology, Maharaja Suhel Dev Autonomous State Medical College and Mahrishi Balark Hospitals, Bahraich, IND; 2 Urology, Sawai Man Singh Medical College, Jaipur, IND; 3 Urology, King George's Medical University, Lucknow, IND

**Keywords:** erα, erβ, foreskin, hypospadias, oestrogen receptors

## Abstract

Introduction: The human skin is an important oestrogen-responsive organ containing the oestrogen receptors ERα and ERβ. Penile foreskin may have similar properties; therefore, typifying the expression and cellular location of oestrogen receptors ERα and ERβ in hypospadiac and normal foreskin can expand our knowledge of the aetiology of hypospadias.

Materials and methods: We prospectively analysed foreskin and longitudinally excised tissue of the urethral plate of 52 male patients undergoing hypospadias repair. Sixteen male patients undergoing elective circumcision were used as controls. Based on the ectopic location of the urethral meatus, hypospadias was classified as distal (group A), mid (group B), and proximal (group C). Healthy controls were included in group D. Oestrogen receptors' location and expression were characterised by immunohistochemical analysis of foreskin and urethral plate tissues.

Results: ERα expression in foreskin was intermediate in control and distal and mid-penile hypospadias and weak in proximal hypospadias. Urethral plate ERα expression was intermediate in distal hypospadias and weak in mid-penile and proximal hypospadias. ERβ immunoreactivity was strong in the stratum basale and stratum spinosum, whereas ERα was localised to the stratum basale of the foreskin.

Conclusions: These data demonstrate a significant role of oestrogen and oestrogen receptors in normal penile foreskin and urethral plate development.

## Introduction

Hypospadias is one of the most common congenital anomalies in urology, with an incidence of 1 in 200 to 300 males [1]. It is characterised by an ectopic urethral opening along the ventral aspect of the penis. In the majority of cases, it is associated with penile curvature (chordee) and incompletely developed ventral foreskin [2].

Hypospadias is usually classified into three categories based on the location of the meatus: the distal, mid, and proximal regions. It is a heterogeneous congenital anomaly with various presentations. It may be a mild or severe variant, isolated or associated with other anomalies. The prevalence of hypospadias has been rising over the past 2 to 3 decades [3,4]. Lund et al. reported an annual increase in prevalence of 2.4% [5]. The rising incidence of hypospadias may be related to the exposure to oestrogenic compounds and various environmental pollutants acting as endocrine disruptors because of their oestrogenic and antiandrogenic properties [6-8].

Crescioli et al. showed that oestrogen receptors are present and work in the external genitalia of human male foetuses. They also showed that oestrogen receptors are essential for the signalling of oestrogenic substances. Crescioli et al. showed that oestrogen receptors are present and work in the external genitalia of human male foetuses and that oestrogen receptors are essential for the signalling of oestrogenic substances. They also suggested that feminisation of the external male genitalia due to prenatal exposure to abnormal levels of external oestrogens may result from an inhibitory effect of oestrogens on cell growth [9]. Qiao et al. demonstrated the expression and location of oestrogen receptors α and β in normal foreskin and preputial tissue in patients with hypospadias [10]. ERα and β are crucial for signal transduction of oestrogenic substances in the penile tissue of humans.

We hypothesise that ERα and ERβ are essential for normal foetal penile prepuce and urethral plate development and that the expression of these receptors differs between normal and hypospadias foreskin. Characterising the cellular location and correlation of receptor levels with the severity of hypospadias may improve our understanding of the aetiology of hypospadias.

## Materials and methods

This study was conducted from 2015 to 2016 in the Department of Urology, after obtaining approval from the institute’s ethics committee. Fifty-two patients with a diagnosis of isolated hypospadias aged between 1 and 12 years who underwent hypospadias repair were enrolled in this study. The control group comprised 16 age-matched healthy boys who underwent circumcision. Patients who had previously received hormonal therapy or been operated for hypospadias were excluded from the study. All patients were divided into three groups based on the location of the urethral meatus and disease severity: Group A (distal), Group B (mid) and Group C (proximal penile). The controls were designated as Group D. Written informed consent was obtained from parents/legal guardians prior to the surgery. All patients underwent standard hypospadias repair according to hypospadias severity. Distal-most dorsal prepucial skin tissue and a longitudinally incised tissue of the urethral plate were obtained during surgery and sent to the pathology department for histopathological examination as well as for immunohistochemistry (IHC).

Tissue preparation

All formalin-fixed tissues were embedded in paraffin. 5μm micro sections of paraffin-embedded tissue were kept at 600-700°C temperature in an oven for 30-40 minutes. Dewaxing was performed with xylene, and two changes of 10 minutes each were performed. It was then kept in absolute alcohol for 10 min twice. The slides were then deionized in distilled water for 5 min and washed with peroxidase solution (methanol-97 ml, H₂O₂-3 ml) for 10-15 minutes. Two changes of 5 minutes duration each were performed using Tris buffer. Antigen retrieval was performed by placing the slides in citrate buffer (Retrieval Box) in a de-cloaking chamber, and the temperature was set at 125 ^o^C, after which the slides were kept at room temperature for cooling (20 min). The slides were then washed with Tris buffer, and sections were marked with a PAP pen. Three to four drops of background snipper were applied for 15 minutes. Slides were then washed twice with Tris buffer two times and used for immunochemistry.

Immunohistochemistry

The following antibodies were used: oestrogen receptor alpha (ERα), mouse monoclonal antibody (1:200 dilution; Acris Antibodies, Inc., San Diego, CA), and Estrogen receptor beta (ERβ) monoclonal antibody at a 1:50 dilution (Acris Antibodies, Inc., San Diego, CA). Primary antibodies were applied for 1 hour, and then the slides were washed with Tris buffer. After the application of the polymer for 30 min, the slides were washed with Tris buffer. DAB was applied for 5 min, and the sections were then washed with distilled water. The slides were kept in hematoxylin for 2 min and washed with running tap water for 5 min. Alcohol changes were made two times for 5 min each. Finally, two changes of xylene for 10 min duration were made, and slides were mounted. Positive controls for ERα were breast cancer cells, and ERβ were intestinal mucosa cells.

A quantitative assay was performed based on the percentage of cells stained for the receptor and the intensity of staining. Under 400 X magnification, 100 cells were counted, maximum staining was observed, and the percentage of receptors was calculated as the number of positively stained cells per 100 cells counted. Digital images were taken and the density of nuclear staining was graded on a subjective scale as none (0), weak (1), intermediate (2) or strong (3).

Statistical analysis

All statistical analyses were conducted using IBM SPSS Statistics for Windows, Version 19 (Released 2010; IBM Corp., Armonk, New York, United States). Quantitative data were expressed as mean ± standard deviation (SD). The study population was divided into four groups based on the severity of hypospadias and control status: distal hypospadias (Group A), mid-penile hypospadias (Group B), proximal hypospadias (Group C), and normal controls (Group D). 

## Results

Patient characteristics

A total of 68 male subjects were enrolled in the study, including 52 patients with hypospadias and 16 age-matched controls undergoing elective circumcision. The hypospadias cohort was sub-classified based on the position of the urethral meatus into distal (Group A, n = 34), mid-penile (Group B, n = 13), and proximal (Group C, n = 5) types. The control group (Group D, n=16) subjects with normal genital anatomy. The mean age across the groups was comparable, with no statistically significant difference observed (Group A: 8.1 ± 1.7 years; Group B: 7.8 ± 1.2 years; Group C: 3.0 ± 0.7 years; Group D: 8.3 ± 1.6 years; p > 0.05) (Table 1).

**Table 1:** Baseline characteristics of all groups

Group	Number of Participants (n)	Mean Age (Years ± SD)
Group A – Distal Hypospadias	34	8.1 ± 1.7
Group B – Mid-Penile Hypospadias	13	7.8 ± 1.2
Group C – Proximal Hypospadias	5	3.0 ± 0.7
Group D – Controls	16	8.3 ± 1.6

Oestrogen receptor expression in prepuce and urethral plate tissues

Oestrogen receptor expression varied across the different hypospadias groups and the control group. In the prepuce, the mean ERα expression was highest in the control group (1.6 ± 0.5), followed by distal hypospadias (1.2 ± 0.5), mid-penile hypospadias (1.1 ± 0.6), and lowest in proximal hypospadias (0.6 ± 0.2). A similar pattern was observed for ERβ in the prepuce, with strong expression in controls (2.8 ± 0.5), intermediate expression in distal (1.9 ± 0.7) and mid-penile hypospadias (1.5 ± 0.8), and weak expression in proximal hypospadias (0.6 ± 0.2).

In the urethral plate, ERα expression was intermediate in distal hypospadias (1.2 ± 0.5), lower in mid-penile (0.6 ± 0.3), and weakest in proximal hypospadias (0.4 ± 0.2). Similarly, ERβ levels in the urethral plate followed a decreasing gradient from distal (1.9 ± 0.7) to mid-penile (1.5 ± 0.7) and were lowest in proximal hypospadias (0.6 ± 0.3). These findings demonstrate a consistent reduction in both ERα and ERβ expression in prepuce and urethral tissues with increasing severity of hypospadias (Table 2).

**Table 2:** Comparison between groups

Variables (Mean ± SD)	Group A (Distal Hypospadias)	Group B (Mid-penile Hypospadias)	Group C (Proximal Hypospadias)	Group D (Control)
Prepuce Skin Erα	1.2 ± 0.5	1.1 ± 0.6	0.6 ± 0.2	1.6 ± 0.5
Prepuce Skin Erβ	1.9 ± 0.7	1.5 ± 0.8	0.6 ± 0.2	2.8 ± 0.5
Urethral Plate Erα	1.2 ± 0.5	0.6 ± 0.3	0.4 ± 0.2	
Urethral Plate Erβ	1.9 ± 0.7	1.5 ± 0.7	0.6 ± 0.3	

## Discussion

Hypospadias is likely multifactorial in origin. The role of testosterone is well explained; however, little work has been done on the role of estrogen in the etiology of hypospadias and normal penile development. Virilisation of the genital tubercle results in phallic growth and urethral tubularisation. It is brought about by the androgen signaling cascade, which involves testosterone, conversion of testosterone to dihydrotestosterone, and interaction of both hormones with androgen receptors. This androgen signaling cascade can be inhibited by intrauterine oestrogen exposure, resulting in arrested growth of the genital tubercle in the foetus [11].

The human skin is an important oestrogen-responsive organ, and oestrogen receptors have been described in normal skin. ERβ is the major receptor expressed in normal human skin [12]. Hypospadias is characterised by dorsal hooding and ventral foreskin deficiency; therefore, the expression pattern of oestrogen receptors in hypospadias and the correlation between oestrogen receptors and the severity of hypospadias may provide insight into the aetiology of hypospadias.

Our study reveals that the normal prepuce foreskin expresses ERα and ERβ receptors. Our results are similar to those of previous studies by Qiao et al., which stated that the expression of ERβ is strong, while ERα expression is weak in normal foreskin [10]. ERβ immunoreactivity is mainly observed in the nuclear compartment of keratinocytes in the stratum basale and stratum spinosum, whereas ERα is localized in the stratum basale layer of the foreskin epidermis.

The levels of ERα and ERβ were lower in hypospadiac foreskin compared to normal foreskin in our study. These levels dropped even more as hypospadias got worse. The difference in foreskin ERα expression was statistically significant between the distal and proximal hypospadias, but not between the distal and mid-penile hypospadias. Also, there was a statistically significant difference in foreskin ERβ expression between the distal and proximal hypospadias and between the mid-penile and proximal hypospadias. However, there was no difference between the distal and mid-penile hypospadias. This is in contrast to a previous study by Qiao et al., which stated that the differences in ER receptor expression did not reach statistical significance [10]. This pattern of reduced expression with the severity of hypospadias indicates that oestrogen receptors, particularly ERβ, play an important role in normal foreskin development.

To the best of our knowledge, this is the first study to evaluate ERα and ERβ receptor expression in the urethral plate tissue of hypospadias patients undergoing hypospadias repair. Our study revealed that the expression of urethral plate ERβ was stronger than that of ERα. The expression of both urethral plate ERα and ERβ decreased with increased disease severity. Crescioli et al. demonstrated oestrogen receptors in the glans, urethra, and corpus cavernosum of human foetal penile tissue [9]. Qiao et al. reported strong ERβ immunostaining in the corpus spongiosum, corpus cavernosum, and penile skin of human foetal peniles [10].

Embryologically, normal urethral development involves midline fusion of the urethral folds. Hypospadias results from arrested development during intrauterine growth. The abnormal urethral plate seen in hypospadias can be thought of as the result of the urethral folds and corpus spongiosum failing to fuse together in the middle. In a mouse model, in utero exposure to oestrogen resulted in developmental arrest of urethral tissue [13]. Taken together with our findings, these results of decreased expression of urethral plate ERβ and ERα in hypospadias boys suggest that oestrogen and oestrogen receptors must be involved in normal urethral plate development in male foetuses.

Limitation

The limitation of our study is that we could not calculate urethral plate ERα and ERβ in normal human penile tissue. Additionally, we did not study events at the critical time of urethral malformation and external genitalia development. We evaluated boys of similar age groups to overcome differences that may be ascribed to postnatal hormonal changes.

## Conclusions

This study highlights a significant reduction in the expression of oestrogen receptors ERα and ERβ in both the prepuce and urethral plate tissues of patients with hypospadias, particularly in more severe forms. The consistent decrease in receptor expression from distal to proximal hypospadias suggests a potential role of impaired oestrogen signaling in the pathogenesis of the condition. ERβ, in particular, showed strong expression in normal tissues and a marked decline with increasing severity of hypospadias, indicating its critical involvement in normal penile and urethral development. These findings support the hypothesis that disruptions in oestrogen receptor-mediated pathways may contribute to abnormal genital development and offer a potential molecular target for further research and therapeutic intervention.
